# Oral feeding in postoperative pancreatic fistula after pancreatoduodenectomy: meta-analysis

**DOI:** 10.1093/bjsopen/zrac099

**Published:** 2022-08-23

**Authors:** James M Halle-Smith, Rupaly Pande, Sarah Powell-Brett, Samir Pathak, Sanjay Pandanaboyana, Andrew M Smith, Keith J Roberts

**Affiliations:** Hepatobiliary and Pancreatic Surgery Unit, Queen Elizabeth Hospital Birmingham, Birmingham, UK; Hepatobiliary and Pancreatic Surgery Unit, Queen Elizabeth Hospital Birmingham, Birmingham, UK; College of Medical and Dental Sciences, University of Birmingham, Birmingham, UK; Hepatobiliary and Pancreatic Surgery Unit, Queen Elizabeth Hospital Birmingham, Birmingham, UK; College of Medical and Dental Sciences, University of Birmingham, Birmingham, UK; Hepatobiliary and Pancreatic Surgery Unit, Leeds Teaching Hospitals NHS Foundation Trust, Leeds, UK; Hepatobiliary and Pancreatic Surgery Unit, Newcastle Upon Tyne Teaching Hospitals NHS Foundation Trust, Newcastle upon Tyne, UK; Hepatobiliary and Pancreatic Surgery Unit, Leeds Teaching Hospitals NHS Foundation Trust, Leeds, UK; Hepatobiliary and Pancreatic Surgery Unit, Queen Elizabeth Hospital Birmingham, Birmingham, UK; College of Medical and Dental Sciences, University of Birmingham, Birmingham, UK

*Dear Editor*

The benefits of early oral feeding and omitting nasogastric drainage within enhanced recovery after surgery pathways are increasingly recognized after pancreatoduodenectomy (PD), where it is associated with earlier mobilization, reduced duration of hospital stay and complications such as delayed gastric emptying (DGE)^1^. Indeed, a recent meta-analysis comparing early oral feeding to enteral and total parenteral nutrition (TPN) after PD showed no difference in postoperative pancreatic fistula (POPF) or DGE rates, as well as a reduced duration of hospital stay in the early oral feeding group^2^; however, POPF is common after PD and many surgeons consider stopping oral feeding over fears that it may stimulate pancreatic exocrine secretion, exacerbate POPF and its related complications (such as post-pancreatectomy haemorrhage) and prolong POPF healing time. Therefore, the aim of this meta-analysis was to investigate the effects of oral feeding upon patients who developed POPF after PD.

A systematic review, performed in line with PRISMA guidelines, identified randomized clinical trials, where oral feeding was compared with standard care, such as fasting with nasojejunal (NJ) tube feeding or TPN among patients who developed POPF after PD. The full search strategy is available as *Supplementary material*. Meta-analyses were performed with Revman 5.3, in line with the recommendation of the Cochrane Collaboration.

Of 432 studies screened, two were suitable for inclusion^3,4^ (*Fig. S1*). Patients who developed POPF after PD were split into two groups, one was fed orally and the other according to standard care, which was TPN in one study and NJ feeding in the other. Oral feeding did not increase the rate of progression to clinically relevant POPF compared with standard care (OR 1.23; 95 per cent c.i. 0.67 to 2.26; *P* = 0.50) (*Fig. S2*). In addition, oral feeding did not seem to prevent fistula healing, measured as the duration of drain placement (median 27 days (oral feeding) *versus* 26 days (TPN)) in one study and time to fistula closure rate (median 17 days (oral feeding) *versus* 17 days (enteral feeding)) in the other. These were similar between the groups in both studies (*Table 1*).

**Table 1:** Comparison of outcomes for patients who developed POPF after PD and were either fed orally or via standard care (such as parenteral or enteral tube feeding)

	Oral feeding		Control	
Study	Fujii *et al*.^3^	Wu *et al*.^4^	Fujii *et al*.^3^	Wu *et al*.^4^
**Total POPF**	59	114	59	114
**Number feeding**	30	57	29	57
**CR-POPF**	20 (67)	29 (51)	19 (66)	25 (44)
**Duration of drain placement (days), median (range)**	27 (7–80)	–	26 (7–70)	–
**Time to fistula closure (days), median (i.q.r.)**	–	17 (15–20)	–	17 (16–20)
**30-day fistula closure rate**	–	50 (88)	–	51 (89)
**Post-pancreatectomy haemorrhage**	0 (0)	2 (4)	2 (7)	0 (0)
**Grade B/C DGE**	1 (3)	–	3 (10)	–
**mortality**	0 (0)	–	0 (0)	–
**Duration of hospital stay (days), median (range)**	29.5 (16–88)	–	29 (17–78)	–
**Readmission**	0 (0)	5 (9)	0 (0)	3 (5)

Values are *n* (%) unless otherwise indicated. – indicates not reported. POPF, postoperative pancreatic fistula; PD, pancreatoduodenectomy; CR-POPF, clinically relevant-post operative pancreatic fistula; DGE, delayed gastric emptying.

There is a paucity of clinical trials in this area, but available evidence suggests that oral feeding in POPF does not worsen the severity of POPF or prolong its healing when compared with conventional feeding routes such as TPN or enteral nutrition. Oral feeding seemed to be safe, with no reports of aspiration pneumonia, and feasible.

Among healthy controls, the only feeding route that completely avoids pancreatic exocrine secretion is TPN, as even enteral feeding is associated with an increase in pancreatic exocrine secretion^5^. Despite this, it has been shown previously that enteral feeding is not only safe in POPF but is also associated with increased POPF closure rates and shorter time to closure compared with TPN^6^. This meta-analysis suggests that oral nutrition is safe, feasible and does not exacerbate POPF or its complications. This seems logical, as the two main physiological stimuli of pancreatic exocrine secretion, cholecystokinin from the duodenum and autonomic nerves surrounding the gastroduodenal and inferior pancreaticoduodenal arteries, are disrupted during PD. A further important physiological mechanism in this population is the ileal brake, which slows gut transit and reduces gastrointestinal secretions, including pancreatic enzymes. Owing to the reconstruction performed in PD, ingested food enters the distal small bowel more rapidly compared with the healthy population, inducing the ileal brake.

This meta-analysis is limited by being able to include just two studies, both with small cohort sizes and differing control groups. Randomization also occurred on different postoperative days. Furthermore, given that patients in the control group in the study by Fujii *et al*.^3^ were fed with TPN, some may consider this as a progression to clinically relevant POPF; however, all patients were started on TPN immediately after surgery, regardless of POPF status.

There is a need for further evidence in this important area. For example, the cost effectiveness of early oral feeding *versus* alternative routes is unknown. Oral feeding avoids complications associated with both TPN and enteral tube feeding, the need for replaced blocked/infected/dislodged lines and tubes, specialist dietetic teams and food preparations. Oral feeding though, in the setting of DGE, may be associated with vomiting which risks aspiration pneumonia and prolonging duration of hospital stay. Further clinical trials in this area are desirable.

## Supplementary Material

zrac099_Supplementary_Data

## Data Availability

The data used for this systematic review and meta-analysis are available in the public domain in the published manuscripts of the included studies.
