# Supplementary material for: Oral feeding in postoperative pancreatic fistula after pancreatoduodenectomy: meta-analysis
Source: BJS Open. 2022 Aug 23;6(4):zrac099. doi: 10.1093/bjsopen/zrac099 (PMC9416862; doi:10.1093/bjsopen/zrac099)
Supplement: zrac099_Supplementary_Data [file zrac099_supplementary_data.zip › Supplementary_Table_1.docx]

**Table S1– Overview of methodology for studies comparing oral feeding to standard care in patient with POPF**

| **Author** | **Country** | **Study Type** | **Total POPF** | **Feeding Methodology** | **Standard care methodology** | **Other methodology detail** |
| --- | --- | --- | --- | --- | --- | --- |
| **Fujii et al. 2015** | Japan | Randomised controlled trial, multicentre  (5 centres) | 59 | Food intake was started on POD 6. POD6-9: rice porridge. POD9-13: soft rice. POD14+ solid diet. TPN used to supplement calories | Fasted until drain removal. Parenteral nutrition 24 h/day. Oral water permitted. | Calorie intake measured daily for each patient and equivalent for both arms |
| **Wu et al. 2019** | Taiwan | Randomised controlled trial, multicentre  (2 centres) | 114 | Oral intake increased to calorie target. | Enteral feeding and nothing by mouth until 3 days after drain removal. | All patients had gastro-jejunal tube placed intraoperatively. Calorie intake measured daily for each patient and equivalent for both arms |
