# Supplementary figures and images for: Oral feeding in postoperative pancreatic fistula after pancreatoduodenectomy: meta-analysis
Source: BJS Open. 2022 Aug 23;6(4):zrac099. doi: 10.1093/bjsopen/zrac099 (PMC9416862; doi:10.1093/bjsopen/zrac099)

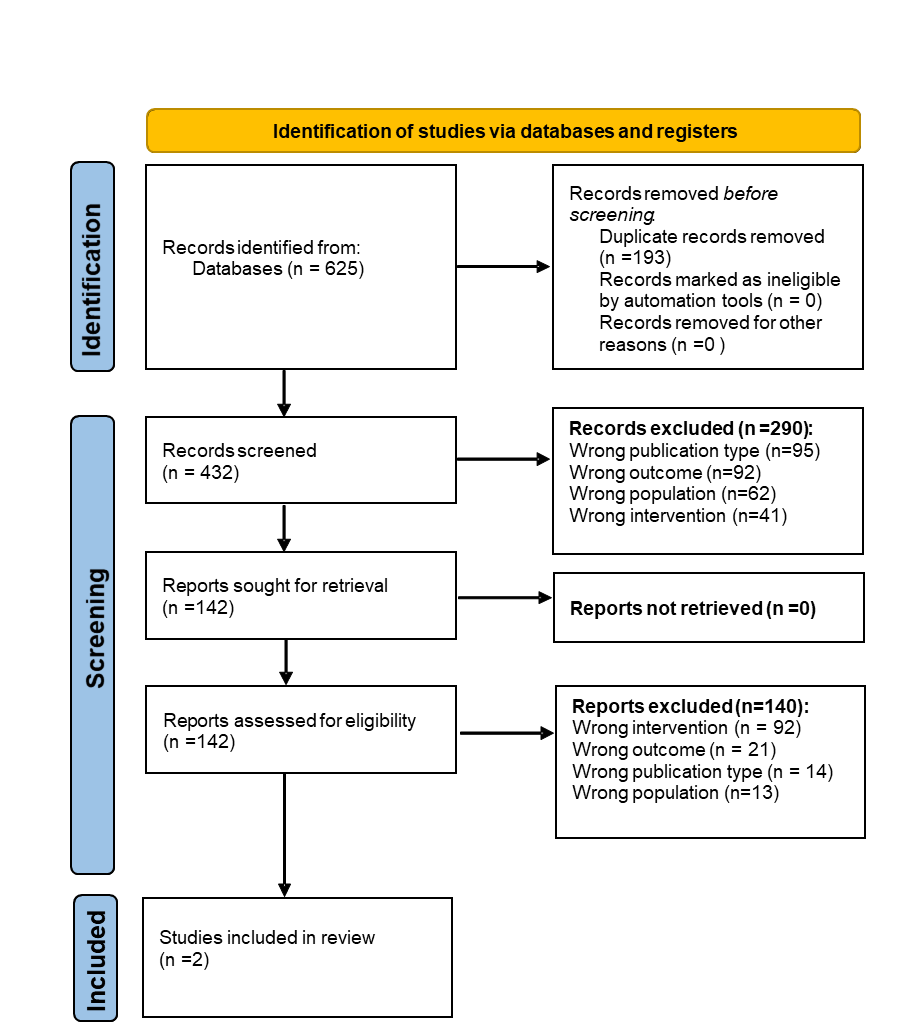

Supplement: zrac099_Supplementary_Data [file zrac099_supplementary_data.zip › Supplementary_Figure_1.tif]

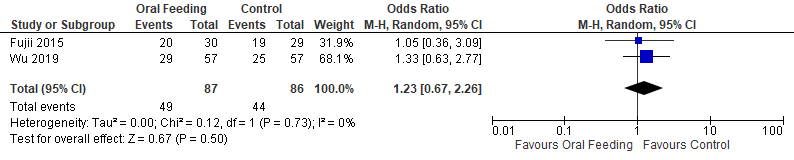

Supplement: zrac099_Supplementary_Data [file zrac099_supplementary_data.zip › Supplementary_Figure_2.tif]

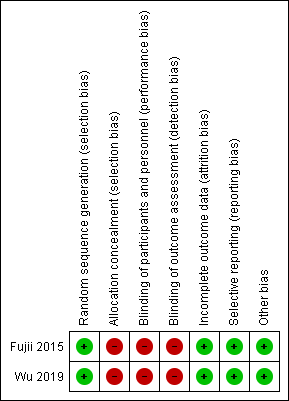

Supplement: zrac099_Supplementary_Data [file zrac099_supplementary_data.zip › Supplementary_Figure_3.tif]
